# Genetic and morphological diversity of *Trisetacus* species (Eriophyoidea: Phytoptidae) associated with coniferous trees in Poland: phylogeny, barcoding, host and habitat specialization

**DOI:** 10.1007/s10493-014-9805-z

**Published:** 2014-04-08

**Authors:** Mariusz Lewandowski, Anna Skoracka, Wiktoria Szydło, Marcin Kozak, Tobiasz Druciarek, Don A. Griffiths

**Affiliations:** 1 Department of Applied Entomology, Faculty of Horticulture, Biotechnology and Landscape Architecture, Warsaw University of Life Sciences (SGGW), Nowoursynowska 159, 02-776 Warsaw, Poland; 2 Department of Animal Taxonomy and Ecology, Faculty of Biology, Adam Mickiewicz University, Umultowska 89, 61-614 Poznan, Poland; 3 Department of Botany, Faculty of Agriculture and Biology, Warsaw University of Life Sciences (SGGW), Nowoursynowska 159, 02-776 Warsaw, Poland; 4 Agrobio, La Mojonera, Almería, Spain

**Keywords:** Cryptic species, Genetic variation, Host specificity, Morphological variation, mtDNA COI, 28S rDNA

## Abstract

Eriophyoid species belonging to the genus *Trisetacus* are economically important as pests of conifers. A narrow host specialization to conifers and some unique morphological characteristics have made these mites interesting subjects for scientific inquiry. In this study, we assessed morphological and genetic variation of seven *Trisetacus* species originating from six coniferous hosts in Poland by morphometric analysis and molecular sequencing of the mitochondrial cytochrome oxidase subunit I gene and the nuclear D2 region of 28S rDNA. The results confirmed the monophyly of the genus *Trisetacus* as well as the monophyly of five of the seven species studied. Both DNA sequences were effective in discriminating between six of the seven species tested. Host-dependent genetic and morphological variation in *T. silvestris* and *T. relocatus,* and habitat-dependent genetic and morphological variation in *T. juniperinus* were detected, suggesting the existence of races or even distinct species within these *Trisetacus* taxa. This is the first molecular phylogenetic analysis of the *Trisetacus* species. The findings presented here will stimulate further investigations on the evolutionary relationships of *Trisetacus* as well as the entire Phytoptidae family.

## Introduction

Mites belonging to the genus *Trisetacus* (Acari: Eriophyoidea: Phytoptidae) are associated with coniferous plants of the families Cupressaceae, Pinaceae and Taxodiaceae, and are widely distributed in Asia, Europe and North America (Xue et al. 2007; Castagnoli et al. 2010; Wang et al. 2012). Coniferous host plants inhabited by these mites are significant in forestry and as ornamental trees, thus many *Trisetacus* species are considered to be serious and economically important pests. They can cause chlorosis, browning, formation of rosette galls (resulting in distorted and stunted needles), cortical galls, inhibition of bud development, as well as destruction of seeds (Castagnoli 1996). Some *Trisetacus* species attack berries and destroy their seeds (Shevchenko 1962) or cause serious damage in nurseries and young stands of cypress (Simoni et al. 2004). The most serious damage is observed when mites occur in large colonies, and mass outbreaks of *Trisetacus* species in forests have been reported (Kruel 1963; Keifer and Saunders 1972; Styer et al. 1972; Shevchenko 1995; Marshall and Clayton 2004). In view of the economic importance of *Trisetacus* mites, damage control and management is imperative, which requires an improvement in the taxonomy of this group (Hoy 2011).

Many coniferous plants provide stable microhabitats in which *Trisetacus* mites proliferate. The evergreen habit and long life span of coniferous trees and their frequent occurrence in monocultures facilitate *Trisetacus* mite dispersal and availability of young tissues on which these eriophyoids feed (Boczek and Shevchenko 1996). The genus *Trisetacus* belongs to the putatively primitive family Phytoptidae (Boczek and Shevchenko 1996; Lindquist 1996), whose members possess some morphological traits that have been recently found in the oldest eriophyoid fossils in Triassic amber (Schmidt et al. 2012). The discovery of eriophyoids in Triassic amber established conifer feeding as an ancestral trait (Schmidt et al. 2012). Thus, high specialization of *Trisetacus* mites on conifers may support their primitive status. It has been found that if insects have elements of conservatism in their food-plant family preferences it can be expected that ancient and phylogenetically basal groups of phytophagous insects are more likely to be associated with ancient plant groups (Ward et al. 2003).

*Trisetacus* species have evolved different associations with their host plants. Many of them use various shelters like buds, seeds, needle sheaths, and bud scales, while others can induce gall formations. Some species have been recorded as vagrants, spending their lives wandering about on needles or twigs (Smith 1984; Castagnoli et al. 2010). However, it has been hypothesized that wanderers in fact may be shelter-associated mites collected during migrations between shelters (Castagnoli 1996). As such, the habitat of some *Trisetacus* species may have been incorrectly described. Some species have been reported as inhabiting more than one type of microhabitat. For example, *Trisetacus juniperinus* (Nalepa) has been found inside buds (causing needle agglomeration, enlargement and necrosis), galls (Jeppson et al. 1975; Łabanowski and Soika 2000; Castagnoli et al. 2010), or inhabiting berries (M. Lewandowski, personal observations). Such habitat differentiation in *T. juniperinus* may suggest that populations occurring in buds and those making galls are distinct, cryptic species (Bouneb et al. 2014; J. Amrine, personal communication). The existence of host races or cryptic species, as has been found in other eriophyoid species (e.g. Skoracka and Dabert 2010; Miller et al. 2013), is therefore suspected in the association of some *Trisetacus* species with several host plants (e.g. *T. relocatus*, *T. silvestris*). Variability in host or habitat use in parasites or herbivores has often been explained by the presence of cryptic species that exhibit narrower microhabitat preference or host range (e.g. Hebert et al. 2004; Steinauer et al. 2007; Johnson et al. 2012). Microhabitat and host specificity have been proposed to play a large role in the speciation of organisms because specialization promotes speciation by reducing gene flow (Futuyma and Moreno 1988; Brooks and McLennan 1993).

The existence of cryptic diversity may confound the taxonomy of a taxon of interest (Bickford et al. 2007). To understand the phylogeny and systematics of the genus *Trisetacus* as well as the family Phytoptidae, which is regarded as a paraphyletic group based almost entirely on plesiomorphic characters, an integrative approach combining morphological and molecular data should be useful (Lindquist and Amrine 1996; de Lillo and Skoracka 2010; Navajas and Navia 2010). Until now, only a few morphometric analyses have been applied to phylogenetic studies of Eriophyoidea (Hong and Zhang 1996a, b, 1997), and few studies have assessed intraspecific variation in Eriophyoidea, including host-adapted strains or cryptic species (Skoracka et al. 2002; Navia et al. 2006; Skoracka and Kuczyński 2006; Magud et al. 2007; Vidović et al. 2010). In recent years, molecular sequence data have become more available to study population genetics, taxonomy, systematics, and evolutionary trends of many acarine groups, including members of the Eriophyoidea (Fenton et al. 1996, 1997, 2000; Kumar et al. 1999, 2001; Navajas and Fenton 2000; Carew et al. 2004, 2009; Lemmetty et al. 2004; Navia et al. 2005; Goolsby et al. 2006; de Lillo and Skoracka 2010; Skoracka and Dabert 2010; Skoracka et al. 2012, 2013, 2014; Miller et al. 2012, 2013). However, we are not aware of any study on phytoptid mites using an integrated morphometric and molecular approach. Among the 644 nuclear and mitochondrial nucleotide sequences obtained for Eriophyoidea and deposited in the GenBank database (Benson et al. 2005), only three represent phytoptid mites, one of which is *T. juniperinus* (http://www.ncbi.nlm.nih.gov/ accessed 22nd October 2013). The combination of genetic and morphological characters to study evolutionary relationships within the genus *Trisetacus* should assist in understanding mite-host relationships and phylogenetic relatedness of species, thereby improving our understanding of Phytoptidae biology, taxonomy and systematics. Discriminating species, especially pest species, is crucial for the development of effective management strategies (Bickford et al. 2007). The misidentification of economically important species hidden within cryptic complexes may lead to serious negative consequences, such as inappropriate diagnoses of parasites and pathogens and ineffective control efforts against pests and invasive species (Armstrong and Ball 2005; Pringle et al. 2005; Bickford et al. 2007).

In this study we link morphometric and molecular techniques in order to examine host and habitat specificity of *Trisetacus* species, viz. *T. juniperinus, T. laricis* (Tubeuf)*, T. piceae* (Roivainen)*, T. pini, T. quadrisetus* (Thomas)*, T. relocatus* Bagnyuk & Shevtchenko, and *T. silvestris* Castagnioli. Specifically we have examined: (1) phylogenetic relationships and interspecific variation within the *Trisetacus* genus, (2) host and habitat specialization of *Trisetacus* species, and (3) the efficiency of two genetic markers (subunit I of the mitochondrial cytochrome c oxidase gene [COI] and D2 region of 28S rDNA) for barcoding *Trisetacus* species.

## Materials and methods

### Sampling

The study included 31 populations of *Trisetacus* species collected from different host plants and microhabitats, and various locations in Poland (Table 1). Mite populations from individual localities, involving collections from the same host, were always gathered from plants 20–400 m apart. Mites were collected from six coniferous species (Pinopsida) belonging to two families, including species important in forestry and ornamental plant nurseries, namely: common juniper, *Juniperus communis* L. (Cupressaceae); European larch, *Larix decidua* Mill.; Norway spruce, *Picea abies* (L.) H.Karst.; Scots pine, *Pinus sylvestris* L.; mountain pine, *Pinus mugo* Turra; and silver fir, *Abies alba* Mill. (Pinaceae).

**Table 1:** Characteristics of the samples used in this study: mite and host plant taxa, mite microhabitats, GPS coordinates of the collection sites, sample codes, number of specimens measured for morphometric study, number of specimens used for DNA sequencing, and associated accession numbers

Mite taxon	Host plant taxon	Mite microhabitat	Geographical coordinates		Sample code	Number of specimens		Accession numbers	
			Lat	Long		Morphometric	Molecular	COI	D2
*Trisetacus juniperinus*	*Juniperus communis*	Galls	53°33′52″N	21°49′28″E	TjunJcomgall1***	30	60	KC776529	–
		Buds	52°55′19″N	23°38′58″E	TjunJcombud1*	30	–	–	–
		Galls	53°33′52″N	21°49′28″E	TjunJcomgall2**	–	60	KC776530	KC776569
		Buds	54°20′49″N	19°14′28″E	TjunJcombud2***	25	30	KC776531	KC776573
		Berries	50°34′28″N	19°29′18″E	TjunJcomberry*	30	–	–	–
		Buds	52°58′37″N	19°44′22″E	TjunJcombud3***	30	30	KC776532	KC776572
		Galls	52°58′37″N	19°44′22″E	TjunJcomgall3***	30	30	KC776533	KC776570
*Trisetacus laricis*	*Larix decidua*	Buds	49°36′47″N	19°31′05″E	TlarLdeca**	–	60	KC776534	KC776564
					TlarLdecb**	–	1	KC776535	KC776563
*Trisetacus piceae*	*Picea abies*	Buds	53°31′30″N	18°07′05″E	TpicPabi**	–	10	KC776536	KC776571
*Trisetacus pini*	*Pinus sylvestris*	Galls	53°31′30″N	18°07′05″E	TpinPsyl1***	30	60	KC776537	–
			51°50′25″N	15°58′12″E	TpinPsyl2a***	}30	60	KC776538	KC776561
					TpinPsyl2b***		60	KC776539	KC776559
			53°46′44″N	21°25′15″E	TpinPsyl3***	30	60	KC776540	KC776560
*Trisetacus quadrisetus*	*Juniperus communis*	Seeds	50°34′28″N	19°29′18″E	TquaJcom1**	–	30	KC776541	KC776568
			52°58′37″N	19°44′22″E	TquaJcom2**	–	30	KC776542	KC776567
*Trisetacus relocatus*	*Picea abies*	Shoot scales	49°17′24″N	20°04′48″E	TrelAalb***	30	60	KC776543	KC776562
	*Abies alba*	Shoot scales	54°20′38″N	22°30′47″E	TrelPabi1*	30	–	–	–
			53°46′44″N	21°25′15″E	TrelPabi2***	30	60	KC776544	KC776566
			49°13′48″N	19°50′10″E	TrelPabi3a***	}30	60	KC776545	KC776565
					TrelPabi3b***		60	KC776546	–
			49°35′31″N	19°31′00″E	TrelPabi4*	30	–		
*Trisetacus silvestris*	*Pinus mugo*	Needle sheaths	49°13′48″N	19°50′10″E	TsilPmug1a***	}30	60	KC776547	KC776557
					TsilPmug1b***		60	KC776548	–
			50°47′26″N	15°34′04″E	TsilPmug2a***	}23	60	KC776549	KC776556
					TsilPmug2b***		60	KC776550	–
	*Pinus sylvestris*	Needle sheaths	53°48′11″N	21°39′08″E	TsilPsyl1*	30	–	–	–
			52°58′41″N	19°44′40″E	TsilPsyl2*	30	–	–	–
			50°02′04″N	18°59′00″E	TsilPsyl3***	26	60	KC776551	KC776555
			52°54′56″N	19°58′51″E	TsilPsyl4***	30	30	KC776552	KC776554
			52°15′29″N	21°16′18″E	TsilPsyl5***	30	10	KC776553	KC776558
Total number of specimens						614	1,161		

* Samples which were used only for morphometric analyses, ** Samples which were used only for molecular analyses, *** Samples which were used for both kinds of analyses

Samples were collected between 2006 and 2011. A single sample consisted of a 15 cm shoot tip, including side shoots collected from the particular host plant and locality (see sample codes in Table 1). Samples were transported to the laboratory, where mite specimens were collected by direct inspection with a stereomicroscope and mounted on slides following standard protocol (Amrine and Manson 1996; de Lillo et al. 2010). Mounted mites were identified as described by Tubeuf (1897), Nalepa (1911), Castagnoli (1973), Jeppson et al. (1975), Bagnyuk (1976), Bagnyuk and Shevtchenko (1982), Keifer et al. (1982), Łabanowski and Soika (2000), and Amrine et al. (2003). One to 60 mite specimens of a given species collected from the majority of samples (see sample codes in Table 1) were placed in labelled 1.5-ml microcentrifuge tubes with 180 μl of ATL buffer (Qiagen, Hilden, Germany) and frozen for further molecular analyses.

### Molecular study

#### DNA extraction, amplification and sequencing

Pooled samples of one to 60 mite specimens stored in ATL buffer were processed as one DNA sample for DNA extraction (Table 1). Isolations were made using a non-destructive method described by Dabert et al. (2008). The voucher specimens are stored in the reference collection of the Department of Animal Taxonomy and Ecology, AMU, Poznań, Poland. To eliminate concern about possible multiple operational taxonomic units within DNA samples extracted from multiple specimens, the precautions described by Skoracka et al. (2012) and Skoracka et al. (2013) were taken. The same DNA samples after extractions were used for amplification of COI and D2 gene fragments.

The cytochrome oxidase subunit I (COI) gene fragment (covering ca. 670 bp of the 5′ terminus of COI gene) was amplified by polymerase chain reaction (PCR) using the degenerate primers bcdF01 and bcdR04 (Skoracka and Dabert 2010). The PCR was carried out in 10 μl reaction volumes containing 5 μl Type-it Multiplex PCR Master Mix (Qiagen), 10 pmol of each primer and 4 μl of DNA template. Amplification of the D2 region of 28S rDNA was performed with the primers D1D2fw2 (Sonnenberg et al. 2007) and 28Sr0990 (Mironov et al. 2012) with the same reaction volume and substrate proportions like COI fragment except that 5 pmol of each primer was used. The thermo-cycling profile of both reactions consisted of one cycle of initial denaturation for 5 min at 95 °C, followed by 35 cycles of denaturation for 30 s at 95 °C, annealing for 30 s at 50 °C, and extension for 1 min at 72 °C; with a final strand extension of 15 min at 72 °C. After amplification, 3 μl of the PCR product was diluted two-fold with distilled water and analysed by electrophoresis in a 1 % agarose gel. Samples containing visible bands were directly sequenced in both directions using 1 μl of the PCR product and 50 pmol of the corresponding sequencing primer (viz. bcdF01 and bcdR04 for COI sequences, and D1D2fw2 and 28Sr0990 for D2 sequences). Sequencing was performed with BigDye Terminator v3.1 using the manufacturer’s protocol and products of the sequencing reaction were analyzed with an ABI Prism 3130XL or 3730 Analyzer (Applied Biosystems, Foster City, CA, USA). Trace files were checked and edited using MEGA5 (Tamura et al. 2011). All sequences have been deposited in the GenBank under the accession numbers indicated in Table 1.

#### Sequence and phylogenetic analyses

*Nalepella brewerianae* Domes 2005 (Phytoptidae) (GenBank accession numbers: KJ410020 for mtDNA COI and KJ410021 for 28S rDNA D2) was chosen as an outgroup species for molecular analyses. The COI and D2 sequences were aligned separately by CLUSTAL W using MEGA5 (Tamura et al. 2011) with default gap weighing parameters, followed by manual adjustment. Alignment of the COI sequences covering 605 bp was translated into amino acids to exclude the presence of pseudogenes or stop codons. The uncorrected pairwise genetic distances (%) were calculated using MEGA5 (Tamura et al. 2011). This simple distance measure was implemented to achieve reliable estimates of both intra- and interspecific genetic variation. Standard error estimates were obtained using a bootstrap procedure (1,000 replicates).

The best-fit models of nucleotide substitution were selected with jModelTest ver. 2 (Darriba et al. 2012; Guindon and Gascuel 2003) based on likelihood scores for 88 different models, Akaike Information Criterion (AIC) and Bayesian Information Criterion (BIC). Two analyses were performed for the COI data set; one analysis (complete) included all sequences available, while the second analysis (reduced) included sequences obtained for mite populations that were also analysed morphologically (see Table 1). The result of the second analysis was used to graphically compare the genetic and morphological variation between and within *Trisetacus* species. For the complete COI data set, TIM1 + I + G (Posada 2003) was chosen as the model for the maximum likelihood (ML) analysis according to AIC, where the proportion of invariable sites (p-inv) = 0.4160 and the gamma distribution shape parameter (G) = 1.0680. The estimated base frequencies were: A = 0.2061, C = 0.1209, G = 0.1540 and T = 0.5190 and substitution rates were [AC] = 1.0, [AG] = 12.6865, [AT] = 2.8725, [CG] = 2.8725, [CT] = 7.0961, [GT] = 1.0. The GTR + I + G model (Rodriguez et al. 1990) was chosen for the Bayesian inference of phylogeny (BI) for the COI complete data. Estimated base frequencies were: A = 0.2008, C = 0.1306, G = 0.1572, and T = 0.5115, with substitution rates [AC] = 0.9710, [AG] = 12.2072, [AT] = 3.1656, [CG] = 1.6401, [CT] = 6.0245, [GT] = 1.0, the proportion of invariable sites was (p-inv) = 0.4230 and the gamma distribution shape parameter was (G) = 1.23. For the reduced data set only BI analysis was performed using HKY + I + G model (Hasegawa et al. 1985), where the transition/transversion ratio was (ti/tv) = 2.3349 the proportion of invariable sites (p-inv) = 0.4440 and the gamma distribution shape parameter (G) = 1.0120. The estimated base frequencies were: A = 0.2101, C = 0.1241, G = 0.1599 and T = 0.5059.

For the D2 28S rDNA data set, the HKY + G substitution model (Hasegawa et al. 1985) was chosen according to BIC, with the following estimated base frequencies: A = 0.2621, C = 0.1649, G = 0.2453, and T = 0.3277. The transition/transversion ratio was (ti/tv) = 3.1457 and the gamma distribution shape parameter was (G) = 0.4030. The TPM3uf + G model (Kimura 1981; Posada 2008) was selected according to AIC. Estimated base frequencies were: A = 0.2626, C = 0.17121, G = 0.2460, and T = 0.3202, substitution rates [AC] = [CG] = 0.5023, [AG] = [CT] = 5.4107 and [AT] = [GT] = 1.0 and the gamma distribution shape parameter was (G) = 0.4050.

For the combined analysis COI and D2 sequences were concatenated for the mite populations, where COI fragment covered from 1 to 605 nucleotides, and D2 from 606 to 1,262 nucleotides. The BI analysis was performed with the same models as for the separate data sets (i.e. GTR + I + G model for COI partition and HKY + G model for D2 partition). Estimated base frequencies for the GTR + I + G were: A = 0.2008, C = 0.1306, G = 0.1572, T = 0.5115, proportion of invariable sites (p-inv) = 0.4240, gamma distribution shape parameter (G) = 1.2310, and the substitution rates [AC] = 0.9720, [AG] = 12.2130, [AT] = 3.1675, [CG] = 1.6411, [CT] = 6.0256 and [GT] = 1.0. The parameters of HKY + G model were the same as to those for D2 dataset analysis.

Bayesian inference of phylogenetic relationships was performed with MrBayes v.3.2 (Ronquist et al. 2012). For each dataset, two independent runs were performed and each consisted of four chains with a number of generations developed until the average standard deviation of split frequencies was less than 0.01. A 50 % majority consensus trees with posterior probability values was composed out of obtained trees, after excluding the first 25 % of trees produced in the analyses. Maximum likelihood analyses were performed with PhyML 3.0 (Guindon et al. 2010). Analyses were set to optimize branch lengths and the tree topology search method used a nearest neighbour interchange algorithm. For each ML analysis, the Approximate Likelihood Ratio Test (aLRT) (Anisimova and Gascuel 2006) was performed.

### Morphometric study

**Fig. 1:**
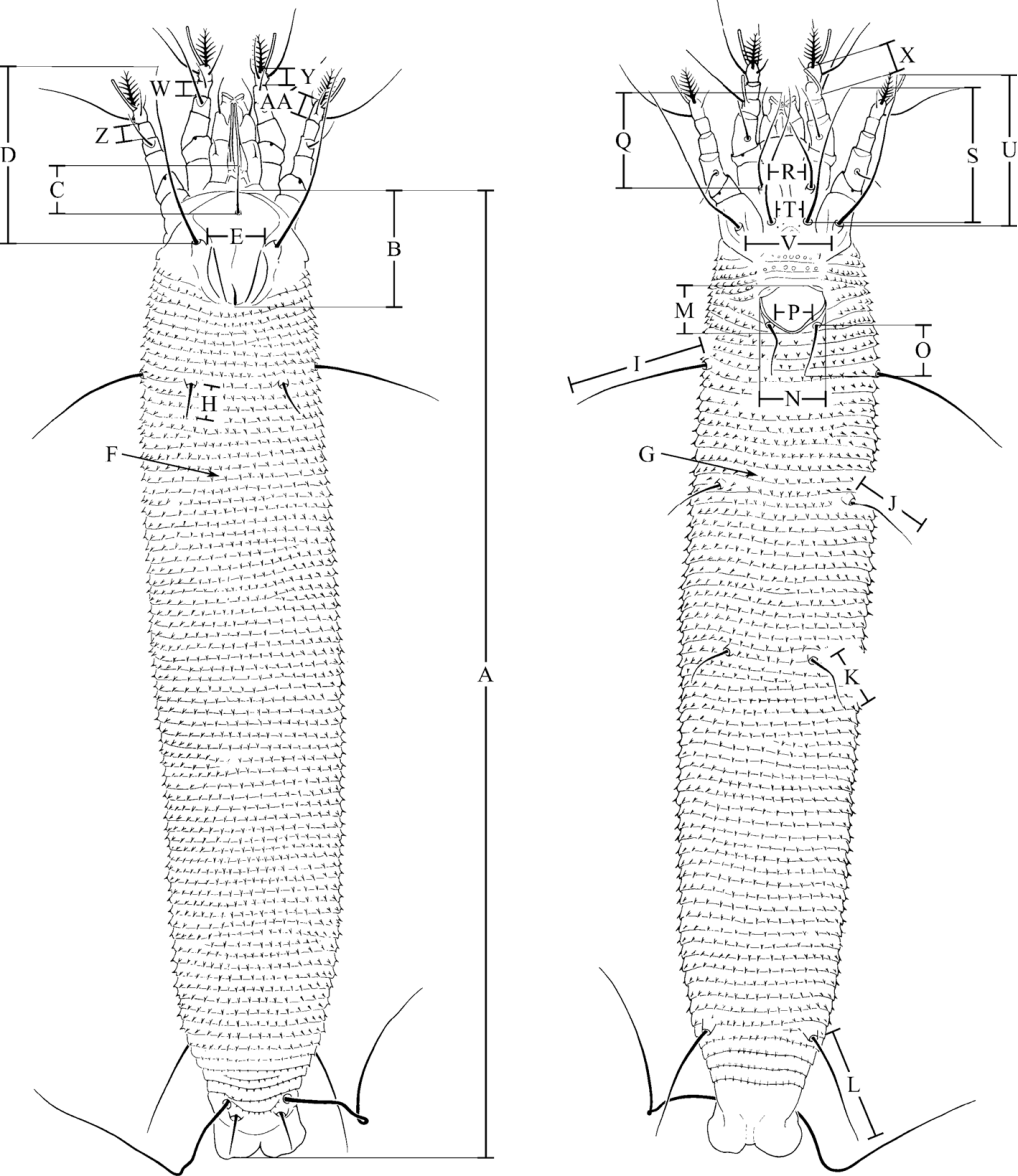
Measurements of *Trisetacus* female morphology used in the morphometric analysis. Explanation of abbreviations: *A* length of body, *B* prodorsal shield length, *C* length of seta *vi*, *D* length of setae *sc*, *E* tubercles *sc* apart, *F* no. of dorsal annuli, *G* no. of ventral annuli, *H* length of setae *c1*, *I* length of setae *c2*, *J* length of setae *d*, *K* length setae of *e*, *L* length of *f*, *M* length of genitalia, *N* width of genitalia, *O* length of setae *3a*, *P* tubercles *3a* apart, *Q* length of setae *1b*, *R* tubercles *1b* apart, *S* length of setae *1a*, *T* tubercles *1a* apart, *U* length of setae *2a*, *V* tubercles *2a* apart, *W* length of tibia I, *X* length of tibial solenidion, *Y* length of tarsus I, *Z* length of tibia II, *AA* length of tarsus II

Sample codes of the *Trisetacus* populations studied morphologically are labelled in Table 1. Twenty-three to 30 females from each population were examined in the dorso-ventral position using a phase-contrast microscope. Twenty-seven morphological traits of each individual were measured with the Soft Imaging System Cell D (Fig. 1).

Discriminant analysis (Krzanowski 2000) was used to explain the variability in the morphology of individuals between various populations inhabiting a particular host taxon (differentiated by locality and microhabitat). The analysis was conducted separately for the four *Trisetacus* taxa (*T. juniperinus*, *T. pini*, *T. relocatus* and *T. silvestris*). This method takes into account the grouping structure due to populations, and helps one to find similar/dissimilar individuals. The values of each trait were first standardized to have mean 0 and variance 1, so that all traits had the same weight in the analysis. The analysis was carried out using the MASS package (Venables and Ripley 2002) in R (R Development Core Team 2013).

Hierarchical cluster analysis was then employed to group the mite populations (described in Table 1) on the basis of their morphology. We used samples for which both morphometric and genetic data were available. The analysis was performed on population mean trait values, which were first standardized to have mean 0 and variance 1. The agglomerative clustering algorithm was carried out using Manhattan distance and the unweighted pair-group average method (Kaufman and Rousseeuw 1990).

Voucher specimens of individual mites measured for the morphometric study are deposited in the reference collection of the Department of Applied Entomology, Faculty of Horticulture, Biotechnology and Landscape Architecture, Warsaw University of Life Sciences (SGGW), Poland.

## Results

### Molecular analyses

#### COI sequence diversity and phylogenetic analyses

The final COI dataset consisted of 26 aligned sequences of 605 bps, representing 25 populations of *Trisetacus* species and one outgroup species. No insertions or deletions were found. In the alignment, 269 (44.5 %) sites were parsimony informative, and 301 (49.7 %) sites were variable. Nineteen haplotypes were identified from 25 COI sequences of *Trisetacus* species with clear correspondence to the host plant species or microhabitat. The average mean divergence over all the sequence pairs (including the outgroup taxon) was 20.6 % (SE = 1.0 %). Pairwise comparison of the COI distances within and between the *Trisetacus* species including outgroup species is given in Table 2.

**Table 2:** Estimates of average divergence (shown as percentages with standard error estimates in parentheses) for mtDNA COI sequence pairs within (emboldened) and between *Trisetacus* species and the outgroup species

	*T. silvestris*	*T. laricis*	*T. relocatus*	*T. piceae*	*T. pini*	*T. quadrisetus*	*T. juniperinus*
*T. silvestris*	8.8 (0.8)						
*T. laricis*	25.5 (1.4)	5.3 (0.9)					
*T. relocatus*	23.2 (1.4)	18.3 (1.3)	9.6 (0.8)				
*T. piceae*	25.1 (1.6)	18.5 (1.4)	17.3 (1.3)	n/c			
*T. pini*	25.4 (1.6)	19.2 (1.5)	18.3 (1.4)	19.0 (1.5)	0.1 (0.1)		
*T. quadrisetus*	24.2 (2.2)	22.4 (1.6)	20.6 (1.4)	20.7 (1.6)	20.3 (1.5)	0.7 (0.3)	
*T. juniperinus*	28.6 (1.5)	23.7 (1.5)	22.4 (1.4)	21.6 (1.4)	22.4 (1.4)	20.4 (1.3)	11.9 (0.9)
*Nalepella brewerianae*	24.8 (1.5)	24.9 (1.6)	25.2 (1.6)	23.1 (1.6)	24.3 (1.6)	22.6 (1.6)	24.0 (1.5)

**Fig. 2:**
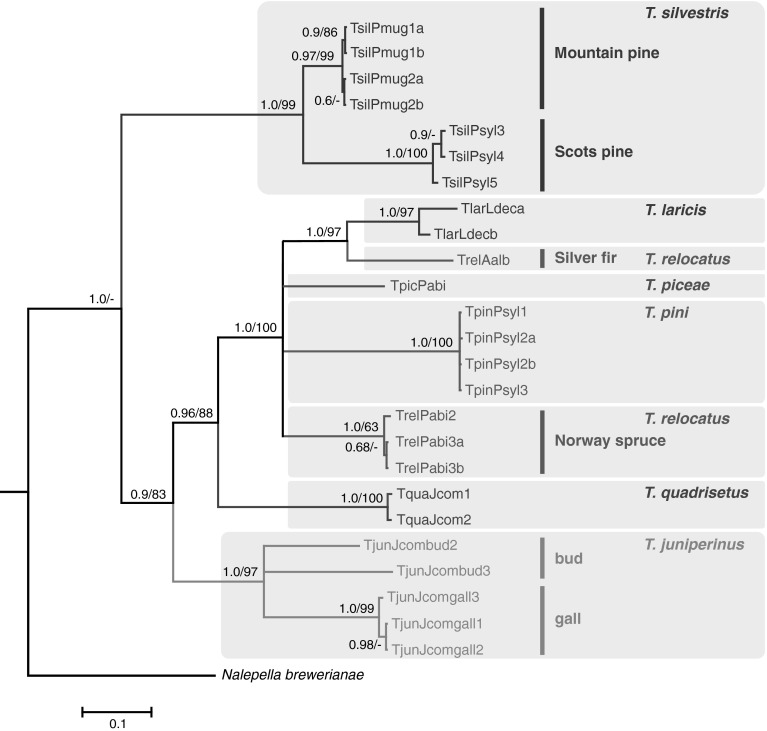
Bayesian inference (BI) tree constructed using the GTR + I + G model on data from the cytochrome c oxidase subunit I sequences of *Trisetacus* and an outgroup species. *Trisetacus* species clades are color coded within *grey boxes*). Within *T. silvestris, T. relocatus*, and *T. juniperinus*, populations associated with different host plant species or habitats are indicated as *vertical lines*. Concordant tree was obtained by Maximum likelihood (ML) analysis, which produced the same topology in defining groups. Statistical supports indicate Bayesian posterior probabilities/maximum likelihood aLRT values. Only statistical supports greater than 0.6/60 are indicated above branches

General topologies of the phylogenetic trees inferred by Bayesian inference (BI) and maximum likelihood (ML) analyses of the nucleotide COI data set were similar and revealed similar structures thus, only the BI tree is presented. It strongly supported the monophyly of the *Trisetacus* genus (Fig. 2). There was also strong support for the monophyly of *T. silvestris*, which clustered separately from the other *Trisetacus* species. Populations of this species were variable and formed two different highly supported clades (distances between clades = 14.9 %; SE = 1.3 %), one containing Scots-pine-associated populations and the second comprising populations associated with mountain pine (Fig. 2). Analyses strongly supported the monophyly of *T. juniperinus*, *T. quadrisetus*, and *T. pini*. Populations of *T. juniperinus* were highly variable (intra-clade variation 11.9 %), with gall-associated populations forming a strongly supported clade, whereas bud-associated populations did not form a distinct clade (Fig. 2). Variation among bud-associated population was 17.2 % (SE = 2). The distance between gall-associated and bud-associated populations was 16.5 % (SE = 1.3). The relationships between *T. relocatus*, *T. pini*, *T. piceae*, and *T. laricis* were not resolved (polytomy). The monophyly of *T. relocatus* was not supported and host-associated populations of this species were highly variable, with 9.6 % intra-species variation. Spruce-associated populations exhibited low variation (0.6 %, SE = 0.2) and clustered together with strong support. Fir-associated populations clustered to *T. laricis* as a sister lineage (Fig. 2). The divergence between host-associated populations of *T. relocatus* was greater (18.6 %, SE = 1.5) than the distance between fir-associated population of *T. relocatus* and *T. laricis* (17.1 %, SE = 1.5).

#### D2 sequence diversity and phylogenetic analyses

The nuclear sequence data comprised 660 base pairs of the D2 region of the 28S rDNA from 21 *Trisetacus* populations and the outgroup species. The average mean divergence over all sequence pairs (including the outgroup taxa) was 14.6 % (SE = 0.7 %). Pairwise comparison of the distances within and between the D2 sequences of *Trisetacus* species including the outgroup species is presented in Table 3.

**Table 3:** Estimates of average divergence (shown as percentages with standard error estimates in parentheses) for D2 region of 28S rDNA sequence pairs within (emboldened) and between *Trisetacus* species and the outgroup species

	*T. silvestris*	*T. laricis*	*T. relocatus*	*T. piceae*	*T. pini*	*T. quadrisetus*	*T. juniperinus*
*T. silvestris*	0.2 (0.1)						
*T. laricis*	10.1 (1.2)	0.3 (0.2)					
*T. relocatus*	9.4 (1.1)	3.9 (0.6)	2.5 (0.5)				
*T. piceae*	10.4 (1.2)	5.6 (0.8)	4.8 (0.7)	n/c			
*T. pini*	11.2 (1.3)	5.9 (0.8)	5.8 (0.8)	7.5 (0.9)	0.0 (0.0)		
*T. quadrisetus*	11.9 (1.3)	14.7 (1.4)	14.6 (1.4)	15.5 (1.4)	15.5 (1.5)	0.0 (0.0)	
*T. juniperinus*	18.1 (1.5)	18.7 (1.5)	18.6 (1.4)	20.7 (1.5)	21.0 (1.5)	21.7 (1.6)	4.5 (0.6)
*Nalepella brewerianae*	38.8 (2.2)	38.4 (2.2)	38.8 (2.2)	38.7 (2.2)	37.0 (2.2)	40.0 (2.2)	40.5 (2.0)

**Fig. 3:**
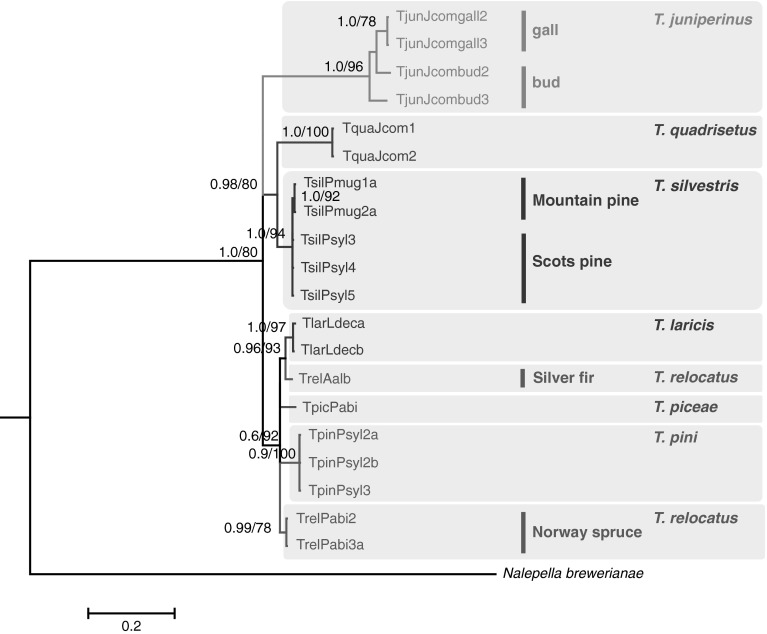
Bayesian inference (BI) tree constructed using the HKY + G model on data from D2 region of 28S rDNA sequences of *Trisetacus* and an outgroup species. *Trisetacus* species clades are color coded within *grey boxes*. Within *T. silvestris, T. relocatus*, and *T. juniperinus*, populations associated with different host plant species or habitats are indicated as *vertical lines*. Concordant tree was obtained by Maximum likelihood (ML) analysis, which produced the same topology in defining groups. Statistical supports indicate Bayesian posterior probabilities/maximum likelihood aLRT values. Only statistical supports greater than 0.6/60 are indicated above branches

General topologies of the phylogenetic trees inferred by Bayesian inference (BI) and maximum likelihood (ML) analyses were similar and consistently revealed the same structure, thus only the BI data is presented. Similar to COI analyses, D2 analyses confirmed the monophyly of *Trisetacus* genus and five *Trisetacus* species with strong support, but did not confirm the monophyly of *T. relocatus*. Spruce-associated populations of *T. relocatus* clustered together in a highly supported clade, whereas fir-associated *T. relocatus* clustered together with a *T. laricis* clade (Fig. 3). Divergence in the D2 gene among the host-associated populations of *T. relocatus* was higher (3.7 %, SE = 0.7) than the distance between fir-associated population of *T. relocatus* and *T. laricis* (2.9 %, SE = 0.6). There was high variation in the D2 gene within *T. juniperinus* (Fig. 3), with divergence between gall-associated and bud-associated populations of 5.3 % (SE = 0.7). Host-dependent variation in *T. silvestris* in the D2 gene was not as evident as in the case of the mitochondrial COI gene. Populations from different pine species clustered together, although mountain pine-associated mites formed a well-supported internal clade (Fig. 3). Divergence between host-associated populations of *T. silvestris* was 0.3 % (SE = 0.2).

### Combined analysis

**Fig. 4:**
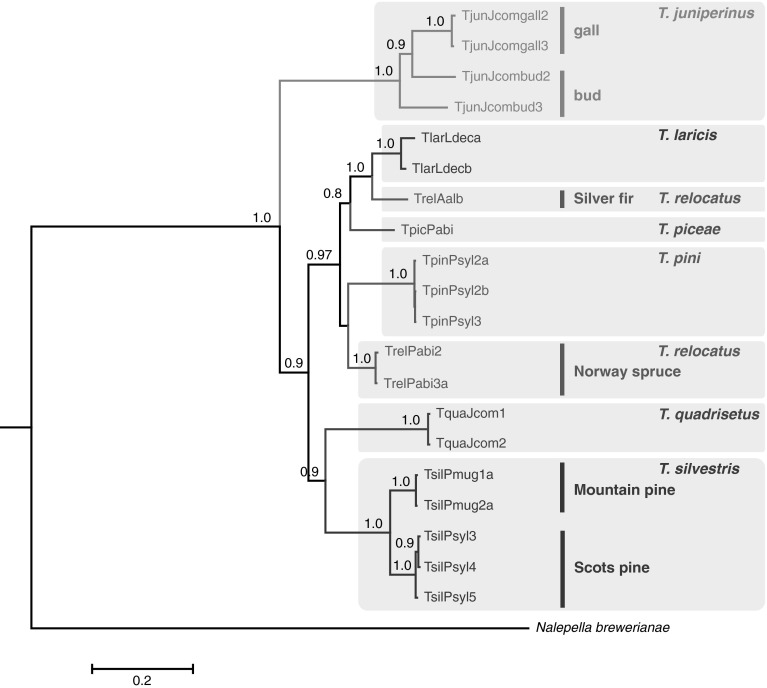
Combined Bayesian inference (BI) tree calculated from the cytochrome c oxidase subunit I sequences (COI) and D2 region of 28S rDNA sequences of *Trisetacus* and an outgroup species. *Trisetacus* species are color coded within *grey boxes*. Within *T. silvestris, T. relocatus*, and *T. juniperinus*, populations associated with different host plant species or habitats are indicated as *vertical lines*. Statistical supports indicate Bayesian posterior probabilities. Only statistical supports greater than 0.6 are indicated above branches

The combined analysis, which included unique variants of nucleotide sequences of the mitochondrial cytochrome *c* oxidase subunit I and nuclear D2 region of 28S, supported the results of single analyses indicating the monophyly of the genus *Trisetacus*, as well as five of the studied species (viz. *T. silvestris*, *T. juniperinus*, *T. laricis*, *T. pini*, *T. quadrisetus*). Moreover, the combined analysis corroborated the lack of monophyly for *T. relocatus,* and the sister relationships of fir-associated populations of this species to *T. juniperinus*. High host-associated and habitat-associated intra-species variation within *T. silvestris* and *T. juniperinus*, respectively, was also detected (Fig. 4).

### Morphometric analyses

**Fig. 5:**
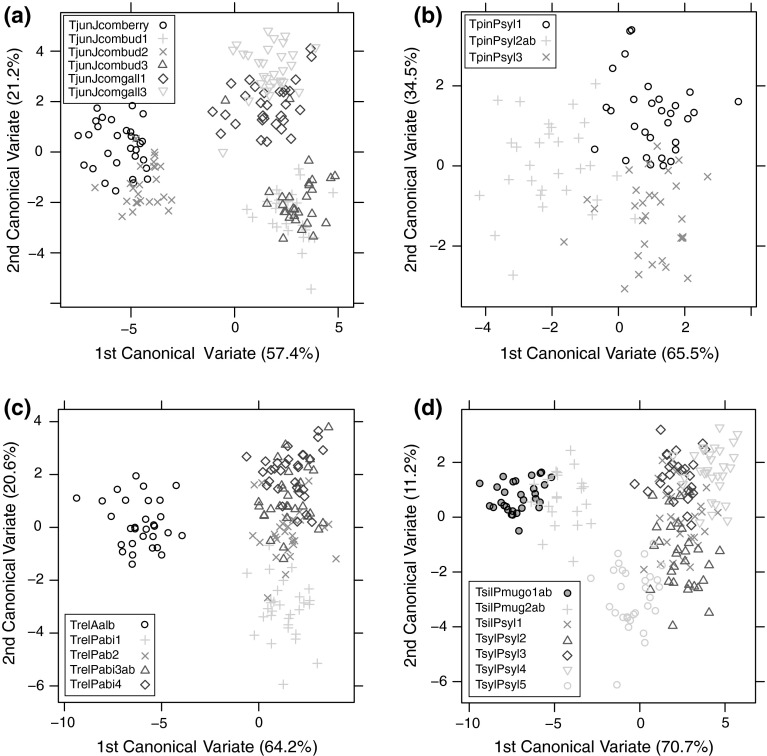
Individuals of *Trisetacus* species plotted against their values for two canonical variates that were calculated using traits listed in Fig. 1. **a** *T. juniperinus*; **b** *T. pini*; **c** *T. relocatus*; **d** *T. silvestris*. Mite population codes are explained in Table 1

The discriminant analysis of 27 traits showed distinct variation between populations of three out of four examined species, viz. *T. juniperinus*, *T. relocatus* and *T. silvestris* (Fig. 5a, c, d). In contrast, there was an overlap of all three populations of *T. pini* relative to canonical variates (Fig. 5b).

Populations of *T. juniperinus* occurring in different microhabitats were significantly distinct from one another, with one exception, viz. the bud-inhabiting population TjunJcombud2 was found to cluster with the berry-associated population (TjunJcomberry) (Fig. 5a). The first canonical variate (explaining 57.4 % of the total variation) clearly separated these last two populations from the other four populations. Individuals from these two populations were characterized by longer opisthosomal setae *d* (J) and *e* (K) (Table 4). The second canonical variate, which showed 21.2 % variation, separated bud-associated and gall-making populations (Fig. 5a). The distance between tubercles of scapular setae (E) and the length of opisthosomal setae *d* (J) contributed most to this discrimination.

**Table 4:** First (CV1) and second (CV2) canonical variable loadings for *Trisetacus* species

Measurement	Canonical variable loadings		Measurement	Canonical variable loadings	
	CV1	CV2		CV1	CV2
*Trisetacus juniperinus*			*Trisetacus pini*		
K	1.17	0.53	J	–0.97	–0.01
E	0.18	–0.84	F	0.04	–0.56
J	–0.69	–0.63	C	0.54	–0.25
G	–0.57	–0.08	S	–0.17	0.46
T	–0.15	–0.54	B	0.07	–0.44
M	0.51	0.17	Z	0.01	0.42
Z	0.51	0.25	X	0.41	0.15
H	0.50	0.51	M	0.15	–0.41
*Trisetacus relocatus*			*Trisetacus silvestris*		
H	–1.43	–0.63	N	–1.09	0.12
V	–1.27	0.31	H	0.90	0.57
A	0.67	0.74	B	–0.83	0.26
X	–0.61	0.73	A	0.38	–0.81
O	–0.72	0.23	E	–0.60	0.43
E	–0.13	–0.56	V	–0.51	0.44
T	0.51	0.28	W	–0.41	–0.49
R	0.45	–0.03	L	0.18	–0.45

See Fig. 1 for abbreviations of measurements

The first canonical variate (identifying 64.2 % of total variability) clearly separated the fir-inhabiting populations of *T. relocatus* from those inhabiting spruce (Fig. 5c). Fir-associated individuals were characterized by longer setae *c1* (H), a greater distance between tubercles of proximal setae on coxosternum II (V) and a shorter body length (Table 4). Spruce-associated populations of *T. relocatus* formed a clumped group with relatively low morphological variability. The exception was one northern population (TrelPabi1), in which the second canonical variate contributed to 20.6 % of the total variation (Fig. 5c). Traits that distinguished this population were linked to body length (A), and lengths of tibial solenidion (X) and setae *c1* (H) (Fig. 5c).

The first canonical variate, describing 70.7 % of the total variability, separated *T. silvestris* individuals inhabiting the host *P. mugo* from *T. silvestris* individuals associated with the host *P. sylvestris* (Fig. 5d). Individuals from *P. mugo* were characterized by wider genitalia (N), a longer prodorsal shield (B) and shorter setae *c1* (H) (Table 4). Mites from *P. mugo* formed a single clade with no distinct differences between populations. The second canonical variate, describing 11.2 % of the total variability, separated the population TsilPsyl5 from populations TsilPsyl4 and TsilPsyl3 (Fig. 5d), thus identifying a difference in body length (A) and length of setae *c1* (H) between them.

### Morphological and COI phylogenetic analyses

**Fig. 6:**
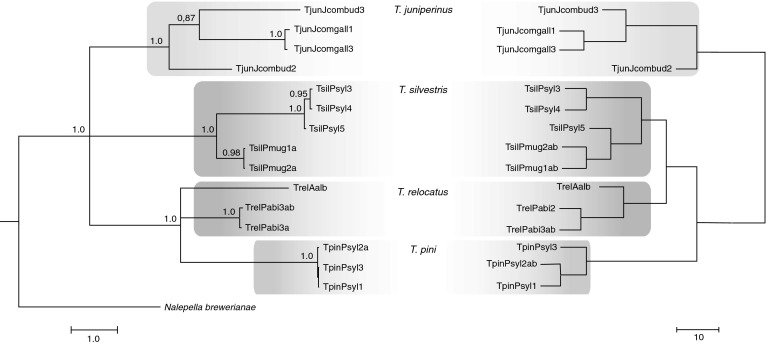
Comparison of a Bayesian inference (BI) tree (with Bayesian posterior probabilities greater than 0.6 indicated above the branches) of the cytochrome c oxidase subunit I (only populations for which morphometric data were available are included) (*left*) with another obtained using Manhattan distance and UPGMA method calculated for morphometric data (*right*)

In general, morphological relationships among *Trisetacus* populations were similar to those revealed by the mtDNA COI, although some differences were observed (Fig. 6). First, the relationships between two host-associated clades of *T. relocatus* and the clade of *T. pini* inferred on the basis of mitochondrial COI sequences were not resolved (polytomy). On the equivalent morphological tree, however, the two *Trisetacus* species clustered separately. Second, three populations of *T. pini* on the basis of mitochondrial COI sequences formed one clade with low variation (Table 2), but morphologically the population TpinPsyl3 was slightly different from the other two populations. Third, on the mitochondrial COI phylogenetic tree, populations of *T. silvestris* clustered into two host-associated clades, whereas on the morphological tree one Scots pine population, viz. TsivPsyl5, clustered together with the populations associated with mountain pine. Fourth, on the morphological tree *T. juniperinus* clustered separately from all other species, whereas the other three species showed the following relationships: *T. relocatus* formed one clade with *T. silvestris*, which was a sister clade to that composed of *T. pini*. On the mitochondrial phylogenetic tree *T. relocatus* formed one unresolved clade with *T. pini*, whereas *T. silvestris* and *T. juniperinus* clustered in distinct clades (Fig. 6).

## Discussion

DNA-based techniques such as DNA barcoding, i.e. taxon identification through analysis of sequences from standardized DNA regions, has recently received much attention (e.g. Hebert et al. 2003; Valentini et al. 2009a, b). Sequences of both mtDNA COI and nuclear D2 as markers for species identification (Hebert et al. 2003; Sonnenberg et al. 2007) were efficient in discriminating between six of the seven *Trisetacus* species tested in this study, viz. *T. silvestris, T. pini*, *T. quadrisetus*, *T. juniperinus*, *T. laricis*, and *T. piceae*. The divergence between these species is comparable to or higher than estimates of interspecific variation in other mite taxonomic groups (e.g. Anderson and Morgan 2007; Dabert et al. 2008; Tixier et al. 2008; Martin et al. 2010; Roy et al. 2010; Skoracka and Dabert 2010; Niedbała and Dabert 2013). Especially high distance values in the D2 region, which is often highly conserved (Lee and O’Foighil 2004), indicate an ancient evolutionary origin of *Trisetacus* species.

The monophyly of most *Trisetacus* species studied here, viz. *T. pini*, *T. silvestris, T. quadrisetus*, *T. juniperinus*, and *T. laricis*, was supported. *Trisetacus piceae* was classified as expected on the basis of COI and D2 but was represented by only one sequence. Thus, to test its monophyly additional samples would need to be sequenced.

In the course of this study, *T. pini* represented a genetic lineage with very low intra-clade variation and a morphologically uniform cluster. However, since *T. pini* has been found only on Scots pine in Poland (Skoracka et al. 2005) and we were not able to analyse populations living on other hosts, genetic and morphological differentiation within this species can not be ruled out given that it has also been found on mountain pine, spruce and larch by other researchers (Nalepa 1887, 1911; Shevchenko et al. 1993; Petanović et al. 1996). It is possible that populations of *T. pini* associated with different hosts diverged genetically and morphologically, as it has been already presented for many phytophagous and parasite taxa (e.g. Magalhães et al. 2007; Gwiazdowski et al. 2011; Jorge et al. 2011; Nishimura et al. 2011; Dickey and Medina 2012; Malagnini et al. 2012), including eriophyoid mites (e.g. Skoracka and Dabert 2010; Vidović et al. 2010; Skoracka et al. 2012; Miller et al. 2012, 2013), with *T. silvestris* (this study) among them.

*Trisetacus silvestris* so far has been collected only from *P. sylvestris* in Italy and Poland (Castagnoli 1973; Lewandowski and Kozak 2008; Kozak and Lewandowski 2010). In the course of this study this species was also found on *P. mugo.* Phylogenetic analyses found *T. silvestris* to be a paraphyletic taxonomic group, with high statistical support for monophyly of two host-associated lineages. Estimates of genetic variation between these two lineages are comparable with inter-specific variation in other mite taxonomic groups (see references above). In addition, morphological analysis supported the divergence of populations living on Scots pine and mountain pine. Similarly, considerable variation was detected within *T. juniperinus,* associated with habitat specialization. Gall-associated populations diverged as a morphologically uniform cluster as well as monophyletic lineage with a large genetic distance from bud-associated populations. These morphological, genetic, physiological and ecological differences between lineages suggest the existence of host-specialized races or even cryptic species within both of these *Trisetacus* species. It is known that host-association or habitat-association may act as a prezygotic ecological barrier of gene flow and lead to genetic differentiation (Schluter 2001; Kassen 2002). Host- and habitat-induced specialization is very common in parasitic and phytophagous taxa, e.g. avian feather lice (Johnson et al. 2012), malaria parasites (Loiseau et al. 2012), parasitic flatworms (Brooks and McLennan 1993), an acanthocephalan parasite (Steinauer et al. 2007), fleas (Arbiv et al. 2012), beetles (Blair et al. 2005), parasitoid wasps (Kankare et al. 2005), many phytophagous insects (Stireman et al. 2005), and other eriophyoid mites (Miller et al. 2013).

Although gall-associated populations of *T. juniperinus* are suspected to be cryptic species, bud-associated populations of this species exhibited high intra-clade genetic and morphological variation. Two bud populations were morphologically similar to each other, whereas one was closer to a berry-associated population. We were not able to obtain DNA material from specimens inhabiting berries, thus any conclusions explaining the genetic relationships between these three populations, differing in their microhabitats, are not available at this time.

*Trisetacus relocatus* was the only species for which the monophyly was not detected. Two host-associated populations of this species were morphologically and genetically distinct. It is possible that fir- and spruce-associated populations of *T. relocatus* are two different species. Moreover, the fir-associated population was genetically more related to larch-associated *T. laricis* than to spruce-associated populations of the same species. The relationships between two populations of *T. relocatus* and *T. laricis* do not reflect the host phylogeny, since *Larix* (larch) is phylogenetically closer to *Picea* (spruce) (subfamily Pinoideae) than to *Abies* (fir) (subfamily Abietoideae) (Wang et al. 1999; Gernandt et al. 2008; Christenhusz et al. 2011). Similarly, the phylogenies of other *Trisetacus* species in this study (Figs. 2, 3, 4, 6) do not reflect host phylogeny, which may suggest that these *Trisetacus* lineages did not co-evolve with their hosts, rather some host-switching might have occurred (Poulin 2007). On the other hand, the split between Pinoideae and Abietoideae is estimated to have happened in the Jurassic or Early Cretaceous period (Gernandt et al. 2008), whereas eriophyoid fossils in amber have been found in the Triassic period (Schmidt et al. 2012). Thus, the co-evolution of these *Trisetacus* lineages with their hosts is certainly a possibility. To differentiate between these scenarios it would be necessary to make a wider cross-sampling of *Trisetacus* species relative to their world distribution and to their host ranges, and to employ additional molecular tools.

It is clear that proper identification of species, especially cryptic species, is important not only for taxonomy and systematics, but also for applied research, particularly in the case of economically important organisms (Navajas and Navia 2010; Hoy 2011; Benefer et al. 2013; Navia et al. 2013), like *Trisetacus* species. Among the *Trisetacus* species in this study, there are some that may be harmful to their coniferous hosts. For example, *T. pini* causes galls on pine twigs, and the large number of galls can decrease the development of young pine or lead to the death of branches of adult trees (Kruel 1963; Shevchenko et al. 1993; Castagnoli et al. 2010). *Trisetacus laricis* and *T. piceae* can live inside buds leading to their death or may inhibit development of shoots (Roivainen 1951; Smith 1979; Kadono 1981; Bagnyuk 1984). *Trisetacus relocatus* inhabits the bases of the current year’s shoots and may hamper their development (Bagnyuk and Shevtchenko 1982). The existence of host- and habitat-associated races or species within *Trisetacus* species raises questions about biological and ecological differences between them. It has been frequently shown that races or species within complexes may differ for traits such as effect on hostplant physiology, pesticide resistance, invasiveness potential, and capacity for agricultural damage (e.g. Dres and Mallet 2002; Umina et al. 2004; de Barro et al. 2011; Navia et al. 2013). Thus, extending our knowledge on biology and diversity of eriophyoid mites is essential for further development of effective plant protection and management strategies.

The broader sampling of *Trisetacus* species throughout their host and geographical ranges as well as estimation of their DNA diversity on the basis of additional marker loci will be necessary to clarify phylogeny of this genus and seek additional cryptic diversity associated with host- and habitat-specialization. The findings presented here can be an influential contribution to further studies on the phylogeny and systematics of *Trisetacus* as well as the entire Phytoptidae family.
